# Molecular characteristics of early‐onset pancreatic ductal adenocarcinoma

**DOI:** 10.1002/1878-0261.13576

**Published:** 2024-01-03

**Authors:** Silvana Debernardi, Lukasz Liszka, Chara Ntala, Katja Steiger, Irene Esposito, Emanuela Carlotti, Ann‐Marie Baker, Stuart McDonald, Trevor Graham, Branko Dmitrovic, Roger M. Feakins, Tatjana Crnogorac‐Jurcevic

**Affiliations:** ^1^ Centre for Cancer Biomarkers and Biotherapeutics, Barts Cancer Institute Queen Mary University of London UK; ^2^ Department of Pathomorphology and Molecular Diagnostics Medical University of Silesia Katowice Poland; ^3^ St Georges University Hospital London UK; ^4^ Institute of Pathology, School of Medicine and Health Technical University of Munich Germany; ^5^ Institute of Pathology Heinrich‐Heine University and University Hospital of Dusseldorf Germany; ^6^ Centre for Tumour Biology, Barts Cancer Institute Queen Mary University of London UK; ^7^ Department of Pathology and Forensic Medicine Clinical Hospital Center Osijek Croatia; ^8^ Department of Cellular Pathology Royal Free London NHS Foundation Trust UK

**Keywords:** early onset pancreatic cancer, KRAS, p16, p53, SMAD4, tumour heterogeneity

## Abstract

The median age of patients with pancreatic ductal adenocarcinoma (PDAC) at diagnosis is 71 years; however, around 10% present with early‐onset pancreatic cancer (EOPC), i.e., before age 50. The molecular mechanisms underlying such an early onset are unknown. We assessed the role of common PDAC drivers (*KRAS*, *TP53*, *CDKN2A* and *SMAD4*) and determined their mutational status and protein expression in 90 formalin‐fixed, paraffin‐embedded tissues, including multiple primary and matched metastases, from 37 EOPC patients. *KRAS* was mutated in 88% of patients; p53 was altered in 94%, and p16 and SMAD4 were lost in 86% and 71% of patients, respectively. Meta‐synthesis showed a higher rate of p53 alterations in EOPC than in late‐onset PDAC (94% vs. 69%, *P* = 0.0009) and significantly higher loss of SMAD4 (71% vs. 44%, *P* = 0.0025). The majority of EOPC patients accumulated aberrations in all four drivers; in addition, high tumour heterogeneity was observed across all tissues. The cumulative effect of an exceptionally high rate of alterations in all common PDAC driver genes combined with high tumour heterogeneity suggests an important mechanism underlying the early onset of PDAC.

## Graphical abstract

The molecular mechanisms underlying early‐onset pancreatic cancer (EOPC) in patients younger than 50 are unclear. Using 90 primary and matched metastases from 37 EOPC patients, we found that the majority bear alterations in all common PC drivers (*KRAS*, *CDKN2A*, *TP53* and *SMAD4*) and show exceptionally high tumour heterogeneity in the latter three, likely contributing to such an early disease onset.

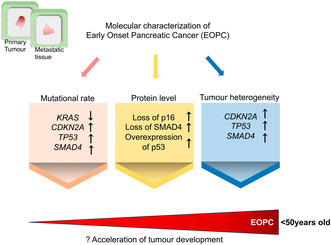

**Abbreviations**
AWM: abdominal wall metastasis
ddPCR: droplet digital PCR
EOPC: early‐onset pancreatic cancer
FFPE: formalin‐fixed, paraffin‐embedded
ICGC: International Cancer Genome Consortium
IHC: immunohistochemistry
IPMN: intraductal papillary mucinous neoplasm
LM: liver metastasis
LNM: lymph node metastasis
LOPC: late‐onset pancreatic cancer
Met: metastasis
MUT: mutation
NOS: not otherwise specified
OM: omental metastasis
PDAC: pancreatic ductal adenocarcinoma
PM: peritoneal metastasis
PT: primary tumour
TNM: tumour, node, metastasis
VCF: variant call file

## 1 Introduction

Pancreatic ductal adenocarcinoma (PDAC) is the fifth most common cause of cancer‐related deaths, with a 5‐year survival rate up to 12% in the US [1]. The median age at presentation is typically 71 years [2]. However, up to 10% of newly diagnosed patients are below the age of 50 years. They are commonly referred as having an early‐onset pancreatic cancer (EOPC) [3, 4]. A significant rise in the incidence of cancers, including PDAC, in young adults, including PDAC, has recently been reported [5].

Epidemiological studies, including our own [6], have shown that EOPC patients are more frequently male and more commonly of Asian or Central and Eastern European origin [5, 7, 8]. They tend to present at a more advanced stage than older patients, receive more aggressive treatment and have slightly better survival [9, 10, 11]. However, overall, the clinicopathological characteristics of EOPC are very similar to those of PDAC in older patients (LOPC, late onset) [6, 12]. Similar to LOPC, risk factors like tobacco, alcohol, obesity and pollution (i.e. environmental exposure to heavy metals and chemicals) have all been associated with PDAC when it presents before age 50 [7, 13, 14]. While pathogenic germline variants, most commonly being BRCA1/2, were reported [15], a relationship of EOPC with hereditary factors and genetic syndromes has not been firmly established [16, 17, 18]. At the genome level, reported differences in mutation frequencies of PDAC driver genes are not consistent [19], and no other definitive genomic, transcriptomic or proteomic differences between EOPC and LOPC have been found [20, 21]. Thus, the detailed molecular mechanisms underlying the reasons for early onset remain to be uncovered.

We previously reported an increased number of PanIN‐1 in EOPC patients [22]. Here, we performed a detailed analysis of the four main PDAC driver genes, i.e. *KRAS*, *TP53*, *CDKN2A* and *SMAD4* [23], to establish if and how their mutational status and their protein expression differ in EOPC and LOPC. We analysed large EOPC cohorts composed mainly of highly relevant cases from Eastern/Central Europe with multiple samples derived from both primary and metastatic lesions. Furthermore, as the growth rate of LOPC is probably slow, with an interval of almost 20 years from its initiation to the development of metastatic clones [24], we also assessed the kinetics of tumour growth in young patients to investigate whether a faster growth rate could explain such early onset of the disease. Finally, as over half of our cases benefited from multiple tissue blocks representing both normal tissue, primary and metastatic tumour, we were also able to investigate tumour heterogeneity.

## 2 Materials and methods

Clinical specimens of 37 EOPC patients collected in the period of last 10 years were obtained from four European Centres (Table S1) with the understanding and written consent of each subject under ethical approval from relevant Institutional Boards. Twenty‐five samples were obtained from the Medical University of Silesia, Katowice, Poland (IRB number: KNW/0022/KB/14/16), seven from the Technische Universität München, Munich, Germany (Ethics approval Reference Number: 403/17 S), three were from Royal London Hospital, London, UK (London Brent Research Ethics Committee Reference Number: 05/Q0408/65) and two from the Pathology department of KBC, Clinical Hospital Centre Osijek, Croatia (R1‐1510/2013).

Histopathological diagnoses of PDAC were established using 2019 WHO criteria [25]. TNM stage was assessed using the American Joint Committee on Cancer (AJCC) staging manual, 8th edition [26]. Multiple formalin‐fixed, paraffin‐embedded (FFPE) tissue blocks were available for 17 patients. In total 90 samples were analysed (Table S1).

### 2.1 FFPE DNA extraction and quality control

FFPE tissues were examined to establish the tumour cellularity: seven EOPC cases from Germany were laser capture microdissected while the remaining samples were macrodissected to achieve cancer cellularity of > 50%. Laser capture microdissection was performed using a Leica LMD6 (Leica Microsystems, Wetzlar, Germany). Briefly, tumour cells were dissected after deparaffinisation and counterstaining with methylene blue. Macrodissection was performed as follows: five 10 μm thick consecutive sections were cut from each sample block, followed by a 4 μm thick section that was stained with Haematoxylin & Eosin to ascertain tumour cell content. The mounted sections were first deparaffinated with two dips in xylene for 3 min each, followed by two dips of 1 min each in 100% ethanol. DNA was isolated using the QIAamp DNA FFPE Tissue Kit (Qiagen, Crawley, UK, 56404). The selected areas were scraped with a blade, transferred in 180 μL of the provided ATL buffer and digested 1.5 h with 20 μL of proteinase K at 56 °C. Subsequently, the samples were incubated for 1 h at 90 °C to reverse the formaldehyde modification of nucleic acids. Washes were performed in the supplied columns, and DNA was eluted in 25 μL of distilled water and quantified using the Quant‐iT PicoGreen dsDNA reagent kit (Invitrogen, Carlsbad, CA, USA, P7589). DNA integrity was assessed by Agilent 2200 TapeStation with D1000 screentape (Agilent Technologies, Santa Clara, CA, USA, PN 5067‐5584) and absolute copy number calculation was performed by quantitative amplification of a 180 bp TaqMan Assay, FHT1 (assay Hs01694011_s1) (Life Technologies, Carlsbad, CA, USA, PN 4331182), using the TaqMan Gene Expression Master Mix (Life Technologies, PN 4369016). Serial dilutions of genomic DNA (Sigma‐Aldrich, Gillingham, UK, Cat. no. 11691112001) of known concentrations were amplified alongside the samples as reference.

### 2.2 Targeted gene sequencing and data analysis

The microfluidic technology from Fluidigm (48.48 Access Array IFC, Fluidigm, South San Francisco, CA, USA, PN AA‐M‐48.48) was used for DNA enrichment and library preparation followed by sequencing with Illumina MiSeq platform (Illumina, San Diego, CA, USA). Thirty target‐specific primer pairs were custom designed using the Fluidigm D3™ assay design tool and GRCh37/hg19 as genome reference (Table S2). All DNA samples underwent preamplification. For the reaction, 100 copies of template DNA were preamplified in a 10 μL reaction mix containing: 1 μL of pooled target‐specific primer pairs (diluted at 500 nm each), and 2.98 μL of preamplification master mix using the FastStart High Fidelity PCR System dNTPack (Roche, Basel, Switzerland, PN 04‐738‐292‐001) following the manufacturer's protocol. Unused primers were eliminated by adding 4 μL of Exo‐SAP‐IT enzyme mix (Affymetrix, Santa Clara, CA, USA, PN 78200) to the reaction mixes and incubating at 37 °C and 80 °C for 15 min each time. Before proceeding to the enrichment amplification, an aliquot of each pre‐amplified product was loaded on Agilent 2200 TapeStation with D1000 screentape for quality control. The Access Array Barcode Library for Illumina Sequencers (Fluidigm, PN 100‐3771) was used to incorporate sample‐specific barcodes and sequencer‐specific adaptor in each amplicon. The PCR products were pooled into one library which was purified using Ampure XP beads and quantified using the Qubit dsDNA high‐sensitivity kit along with the Agilent 2200 Tapestation D1000 Screentape. Sequencing was performed at 150 bp paired end using the MiSeq Reagent Kit v2 (300 cycles) (Illumina, MS‐102‐2002) at QMUL genomics core facility, Genome Centre (http://www.smd.qmul.ac.uk/gc/).

Raw sequence data was processed using the pre‐installed miseq reporter software on the MiSeq instrument (Illumina). The PCR amplicon workflow was followed which includes preparing a manifest file detailing the targets to be used for variant identification. The sample sheet used for processing included the Fluidigm adapter sequences for these to be trimmed prior to alignment. Demultiplexing of the raw base call files to FASTQ format was carried out using the sample IDs and barcode sequences specified by the sample sheet. Sequencing reads were aligned to the human reference genome (GRCh37/hg19) using the Burrows‐Wheeler algorithm (bwa) (SourceForge, San Diego, CA, USA) [27]. Variant calling was performed using the Genome Analysis Toolkit (gatk), producing a single variant call file (VCF) for each sample. The individual VCF files were then combined using vcftools [28]. Mutations with allele frequency ≥ 0.2 were counted. SNPnexus was used for interpretation of nucleotide changes [29]. Sorting intolerant from tolerant variants (SIFT) (https://sift.bii.a‐star.edu.sg/) and polymorphism phenotyping (PolyPhen) (http://genetics.bwh.harvard.edu/pph2/) programmes were also used for the interpretation of identified missense changes [30, 31]. In addition, mutations were checked against the Catalogue Of Somatic Mutation In Cancer (COSMIC) database (https://cancer.sanger.ac.uk/cosmic).

### 2.3 Droplet digital PCR

Droplet Digital PCR (ddPCR) was performed using the ddPCR *KRAS* Screening Multiplex kit (BioRad, Hercules, CA, USA, 186‐3506) and the QX200 Droplet Generator (BioRad). 20 ng of DNA extracted from FFPE was used for each reaction mix, in a final volume of 20 μL, containing 2× ddPCR Supermix for Probes (no dUTP), 20× multiplex primers 9 μm/probes 5 μm (FAM + HEX), 2 U of restriction enzyme MseI (New England Biolabs, Herts, UK) and 2 U of Uracil DNA glycosylate (UDG) (New England Biolabs). 20 μL of mix and 70 μL of droplet generation oil were dispensed in the cartridge for droplet generation, and 40 μL of the generated emulsion was transferred in a PCR plate and amplified according to manufacturer's recommendations.

The multiplex screening kit contains probes and primers to assess the presence of *KRAS* mutations at codons 12 and 13 (G12A, G12C, G12D, G12R, G12S, G12V and G13D). The QX200 Droplet Reader and the quanta soft Software were used for analysis and data interpretation. All the reactions were performed in duplicate.

### 2.4 Immunohistochemical (IHC) analysis

IHC was performed using the Ventana Discovery System XT (Roche Diagnostics Ltd.) at the core Histopathology Laboratory at Barts Cancer Institute using mouse monoclonal anti‐human p16, diluted 1 : 300 (clone 2D9A12, Abcam, Cambridge, UK, ab54210), mouse monoclonal anti‐human p53, diluted 1 : 50 (clone DO‐7, Dako, Glostrup, Denmark, M7001), rabbit monoclonal anti‐human SMAD4, diluted 1 : 1000 (clone EP618Y, Abcam, ab40759) and rabbit monoclonal anti‐human Ki67 (diluted 1 : 3000, clone ERP3610, Abcam, ab92742). Secondary antibody: Biotinylated goat secondary antibody, anti‐mouse IgG, anti‐rabbit IgG (760‐4205, Roche). The diluent Discovery Ab Diluent; 760‐108, Roche, was used. The Streptavidin‐Biotin system was used for detection with diaminobenzidine tetrachloride (DAB) as chromogen substrate was from Discovery DAB D; 760‐4158, Dab Map kit, Roche. The standard antigen retrieval method was Heat Induced Epitope Retrieval (HIER) in Tris‐EDTA buffer pH 7.8 at 95 °C for 44 min (standard CC1; 950‐124, Roche). Staining protocol #107 for Ventana Discovery XT system was used for all four antibodies. Washing between the steps was performed with Reaction buffer (Tris‐based buffer pH 7.6–7.8; 950‐300, Roche), and 100 μL of diluted primary and secondary antibodies were used in the procedure. Paraffin sections of 4 μm thickness were deparaffinised in the Ventana Discovery XT platform using EZ Prep solution (950‐100, Roche) at 75 °C 8 min. Cell Conditioning was performed using Conditioner #1, Standard CC1, at 95 °C for 44 min, and blocking with endogenous peroxidase Inhibitor D (760‐4157, Dab Map Kit, Roche) was done at 37 °C for 4 min. Tissues were then incubated with primary antibody for 60 min at room temperature, and subsequently with biotinylated goat secondary antibody at 42 °C for 20 min, followed by blocking for 4 min at 42 °C with one drop of Blocker D (760‐4161, Dab Map kit, Roche). Peroxidase labelled Streptavidin (HRPO) (Discovery SA‐HRP D, 760‐4162, Dab Map kit, Roche) was applied and tissue left for 16 min at 42 °C, followed by a series of short incubations: twice 8 min at 42 °C, with one drop of DAB/DAB H_2_O_2_ (Discovery DAB/H_2_O_2_ D, 760‐4159, Dab Map kit, Roche); 4 min at 42 °C with one drop of Copper D enhancer (Discovery Copper D; 760‐4160, Dab Map kit, Roche); 8 min at room temperature with Haematoxylin (760‐2021, Roche) for counterstaining; and 8 min with Bluing Reagent (760‐2037, Roche) for post counterstaining. Slides were then cleaned in warm tap water with detergent and dehydrated in graded ethanol and xylene. Coverslips were added in permanent mounting media (Liquid Coverslip, LCS, 650‐010, Roche).

Scoring was performed according to Oshima et al. [32]. Islet cells served as an internal positive control. p53 immunoreactivity was considered to be either ‘lost’, when the neoplastic cells showed a virtual absence of nuclear labelling compared with adjacent normal tissue (immunoreactivity in < 5% of neoplastic cells) (p53 loss) or ‘overexpressed’ when immunostaining showed a robust nuclear accumulation of the protein in ≥ 30% of neoplastic cells compared with adjacent normal cells. For p16, lack of nuclear staining was interpreted as protein loss irrespective of cytoplasmic staining, according to Wilentz et al. [33]. Immunolabelling of SMAD4 was scored as ‘lost’ when no cytoplasmic or nuclear labelling was seen. Normal acinar, ductal, islet and stromal cells in each case served as internal controls for positive SMAD4 immunolabelling.

There are no guidelines on how to report Ki67 expression in PDAC, so WHO 2019 recommendations for Ki67 index assessment in neuroendocrine neoplasms of the digestive system were followed [34], and Ki67 labelling index was defined as the percentage of cancer cells with nuclear Ki67 expression. At least 500 neoplastic cells were manually counted in 1 to 3 high‐power fields in the tumour area with the subjectively highest extent of expression (hot spot). Any nucleus or mitotic figure with visible accumulation of chromogen was scored as positive, irrespective of stain intensity [35]. Scoring was performed using Cell Counter plugin in imagej software [36]. The 10% and 50% cut‐off values were selected as in the study of Pergolini et al. [37]. IHC slides were scored independently by two board‐certified pancreatic pathologists (CN, LL), and discrepant results were scored by consensus.

### 2.5 Meta‐synthesis

Alteration frequencies of each of the four drivers in EOPC and LOPC patients were extracted from the International Cancer Genome Consortium (ICGC) Data Portal Release 28 (https://dcc.icgc.org; Accessed 14 October 2022) [38]. Simple somatic mutations were available for three studies: PAAD‐US (Cancer Genome Atlas programme, TCGA, *n* = 136 donors), PACA‐AU (Australian Pancreatic Cancer Genome Initiative, APGI, *n* = 360 donors) and PACA‐CA (Ontario Institute for Cancer Research, Canada, *n* = 226 donors). DNA somatic mutation and protein expression changes detected by IHC were collected for the two age groups, and Fisher's exact test was applied for the comparative analyses using graphpad prism, version 9 (Dotmatics, Boston, MA, USA).

### 2.6 Tumoural heterogeneity

The tumoural heterogeneity based on somatic DNA mutations was quantified using the Jaccard Index, or coefficient of similarities, as described previously [39]. The Jaccard Index is obtained by calculating the ratio of the shared variants to the total of all variants. For patients with multiple samples, the median of the Jaccard Index of every possible pairwise comparison was taken. The Jaccard Index has a range of 0–1, where 0 represents 100% heterogeneity and 1 is 100% similarity.

The study methodologies conformed to the standards set by the Declaration of Helsinki.

## 3 Results

The basic demographic information, sample details and the performed analyses are summarised in Table S1. The mean patients' age was 43.2 years (range 31–49 years). Of 90 tissue samples used for DNA extraction, 79 samples from 33 patients (22 histologically normal appearing tissues, 39 primary tumours (36 PDAC NOS, one PDAC associated to intraductal papillary mucinous neoplasm (IPMN) and two adenosquamous carcinomas), and 18 metastatic lesions) were sequenced successfully. The full list of mutations, including allele frequencies and the quality score, is reported in Table S3.

### 3.1 Mutational status of KRAS, CDKN2A, TP53 and SMAD4

**Fig. 1:**
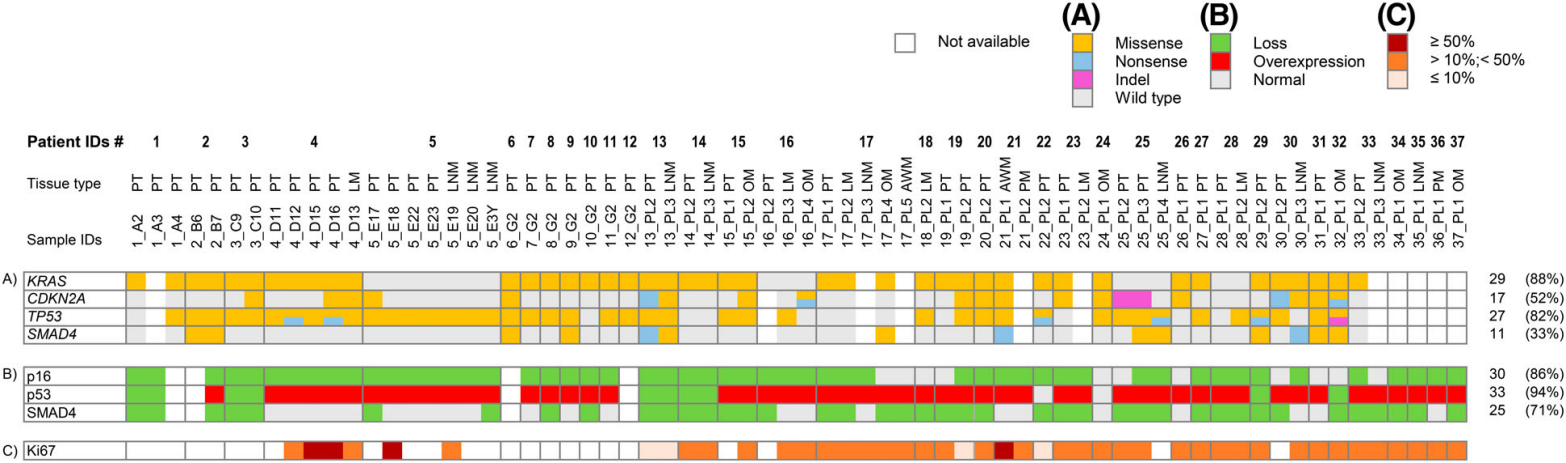
Graphic representation of gene mutations and protein abnormalities. (A) Somatic mutations per gene (row) and per patient (column) obtained by sequencing; (B and C) Immunohistochemistry results. Protein loss and overexpression are colour coded as described in the key. AWM, abdominal wall metastasis; LM, liver metastasis; LNM, lymph node metastasis; OM, omental metastasis; PM, peritoneal metastasis; PT, primary tumour.

A summary of the mutational status is depicted in Fig. 1A.

*KRAS* hotspot mutations at G12/13 and Q61 sites were found in 29 of the 33 (88%) patients when the results from sequencing and ddPCR were combined (Table S4). The most common mutations were *KRAS* ^*G12V*^, found in seven patients and *KRAS* ^*G12D*^ in six. Patient n. 14 had both mutations: the primary tumour carried a G12V, while the metastatic lymph node had an additional G12D mutation. Two patients had multiple *KRAS* variants: patient n. 20 carried a G12V and G13S mutations and patient n. 31 had G12R and G12S mutations (Table S4).

In addition to the activating mutations, five patients also carried less common missense mutations (patients n. 1, n. 3, n. 4, n. 20 and n. 24), and patient n. 5, who was lacking hotspot *KRAS* mutation, carried two missense mutations: T20M in one of the four primary tumours, and T35I in the lymph node metastases (Table S4).

Sixteen patients (48%) carried a total of 27 mutations in *CDKN2A* gene (Fig. 1A): three were nonsense mutations, one was a single nucleotide insertion causing a frameshift, and the remaining were all missense mutations (Table S3). Five of the missense mutations affected a region coding for the less common product known as p12, which is the result of an alternative splice donor site within intron 1. The resulting small protein is exclusively expressed in pancreatic cancer [40].

Twenty‐seven patients (82%) carried *TP53* mutations, with 1–7 mutations per sample (Fig. 1A). Of 59 mutations, six were nonsense, one was a nucleotide insertion causing frameshift and premature protein truncation, the remaining were missense mutations (Table S3). Approximately 77% of the mutations were affecting the DNA binding domain of the protein (exons 5–8).

*SMAD4* was mutated in 11 patients (33%) (Fig. 1A): 21 mutations affected the protein, causing four nonsense and 17 missense amino acid substitutions in the MH2 domain (amino acid 323–552) (Table S3). D493H and W509*, two pathogenic amino acid substitutions associated with pancreatic cancer [41] were found in one and two patients, respectively.

In summary, six of 33 patients (18%) had somatic mutations in all four genes. Around 30% of patients presented somatic mutations in three driver genes and around 40% in two genes. Four patients carried mutations in only one driver gene (12%), three of these had *KRAS* and one had *TP53* mutations (Table 1A).

**Table 1:** Number of mutated genes per patient. (A) Somatic mutation data (33 patients); (B) immunohistochemistry results (35 patients); (C) combination of somatic mutations and immunohistochemistry data (31 patients).

	*N* patients (%)
(A) Somatic mutations	
4 genes	
*KRAS, CDKN2A, TP53, SMAD4*	6 (18%)
3 genes	
*KRAS, CDKN2A, TP53*	6 (18%)
*KRAS, TP53, SMAD4*	3 (9%)
*CDKN2A, TP53, SMAD4*	1 (3%)
Total	10 (30%)
2 genes	
*KRAS, TP53*	7 (21.3%)
*KRAS, CDKN2A*	3 (9%)
*CDKN2A, TP53*	2 (6%)
*KRAS, SMAD4*	1 (3%)
Total	13 (39.4%)
1 gene	
*KRAS*	3 (9%)
*TP53*	1 (3%)
Total	4 (12%)
(B) Immunohistochemistry	
3 proteins	
p16, p53, SMAD4	23 (65.7%)
2 proteins	
p16, p53	7 (20%)
p53, SMAD4	2 (5.7%)
p16, SMAD4	1 (2.8%)
Total	10 (28.5%)
1 protein	
p53	2 (5.7%)
None	1 (2.8%)
(C) Somatic mutations + immunohistochemistry	
4 genes and proteins	
*KRAS, CDKN2A*/p16*, TP53*/p53*, SMAD4/*SMAD4	21 (67.8%)
3 genes and/or proteins	
*KRAS, CDKN2A*/p16*, TP53*/p53	5 (16.1%)
*KRAS, TP53*/p53*, SMAD4/*SMAD4	1 (3.2%)
*CDKN2A*/p16*, TP53*/p53*, SMAD4*/SMAD4	3 (9.7%)
Total	9 (29%)
2 genes and/or proteins	
*CDKN2A*/p16*, TP53*/p53	1 (3.2%)

In total, we analysed 22 histologically normal appearing, matched to tumour, tissues. Amongst these, only one mutation in *KRAS* gene was seen, which was shared by both normal and tumour tissues in patient n. 6; however, this G‐ > A substitution was intronic and therefore silent. *CDKN2A*, in normal tissue, showed two mutations (C‐ > T and G‐ > A) leading to the same synonymous substitution, L117L, which occurred in matched normal/tumour tissues (patient n. 3) and matched normal/lymph node metastasis (patient n. 25). For *TP53* the missense P72R mutation was recorded affecting normal tissue and most matched tumour samples in 11 patients. No mutations in *SMAD4* were seen in matched normal/tumour tissues. So, it appears that normal tissues were not affected by significant mutations.

### 3.2 Immunohistochemistry of p16, p53 and SMAD4

Tissue for immunohistochemical analysis of p16, p53 and SMAD4 was available for 35 patients. Immunohistochemistry results are graphically presented in Fig. 1B.

**Fig. 2:**
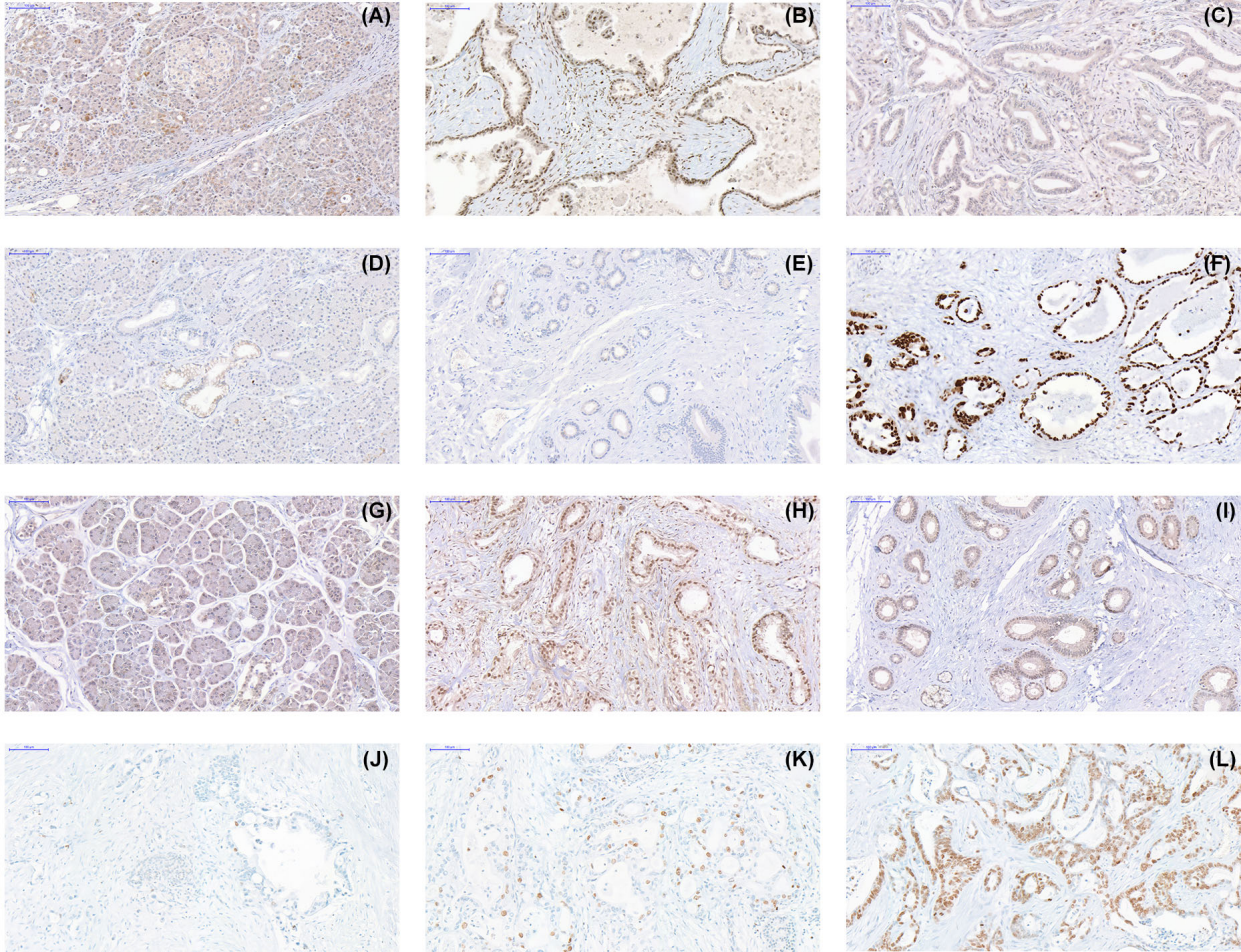
Representative immunohistochemistry images. (A) p16 expression in histologically normal pancreas; (B) primary tumour expressing p16; (C) primary tumour showing loss of p16 expression; (D) histologically normal pancreas expressing p53; (E) primary tumour with loss of p53; (F) primary tumour showing nuclear accumulation of p53; (G) histologically normal pancreas expressing SMAD4; (H) peritoneal metastasis with expression of SMAD4; (I) primary tumour showing loss of SMAD4; (J) primary tumour with ≤ 10% of cells expressing Ki67; (K) primary tumour expressing Ki67 in 10% to 50% of cells; (L) expression of Ki67 in > 50% of neoplastic cells in an abdominal wall metastasis (Scale bars indicate 100 μm).

Loss of p16 immunolabelling was identified in 30 of 35 patients (86%) (Table S5). Representative images are shown in Fig. 2A–C. Abnormal immunolabelling of p53 was detected in 30 of 35 patients (85.7%) (Table S5). Six patients (17%) had a complete absence of p53 immunolabelling. Twenty‐seven (77%) of the immunoreactive cases showed robust nuclear accumulation in ≥30–90% of neoplastic cells. In two patients, nuclear accumulation was present in <20% of neoplastic cells, interpreted as normal (patients n. 22 and n. 24) (Table S5 and Fig. 2D–F).

Loss of SMAD4 immunolabelling was identified in 25 cases (71%) (Table S5 and Fig. 2G–I).

In summary, most patients showed aberrant immunoreactivity (loss or overexpression) for all three proteins (65.7%) and 28.5% had alteration of two proteins. Only one patient (patient n. 24) did not show any protein alteration (Table 1B), although he carried three missense mutations, one in *CDKN2A* and two in *TP53* (Table S3).

Overall, when mutation analysis and IHC data were combined, almost 70% of patients had alterations in all four PDAC drivers and 29% had alterations in three (Table 1C). The results of combined somatic mutations and protein expression alterations per each individual gene/protein are shown in Table 2. *CDKN2A* was affected in 94% of patients, mutations and abnormalities of p53 were present in all, and the majority of patients (82%) had mutations and/or loss of SMAD4 protein.

**Table 2:** Summary results of combined somatic mutations and IHC abnormalities. IHC, immunohistochemistry; MUT, mutations detected by sequencing.

	KRAS	*CDKN2A*/p16		*TP53*/p53			*SMAD4/*SMAD4	
	MUT	MUT	Loss IHC	MUT	Nuclear accumulation IHC	Loss IHC	MUT	Loss IHC
N. of patients and %	29/33	17/33	30/35	27/33	27/35	6/35	11/33	25/35
	88%	52%	86%	82%	77%	17%	33%	71%
MUT + IHC combined		33/35		35/35			28/35	
		94%		100%			80%	

### 3.3 Tumour growth rate

Tissue for Ki67 immunohistochemistry was available for 28 patients. Ki67 expression was present in 27 patients, including 21 primary tumours and 23 metastatic tissues (Fig. 1C). The median percentage of cancer cells with nuclear expression of Ki67 was 25% for primary tumours and 26% for metastatic tissue (Table S5). Only two patients had an index > 50% (Fig. 2L), three (11%) had an index below 10% (Fig. 2J) and the majority of patients (79%) were in the intermediate group with Ki67 immunoreactivity ranging from 11% to 49% (Fig. 2K). The Ki67 index in primary tumours and in metastases was not significantly different.

### 3.4 Meta‐synthesis results

Meta‐synthesis of simple somatic mutations of the four driver genes was performed using the ICGC data from 722 donors. The rate of the mutations did not differ between EOPC and LOPC (Table 3A). The frequency of *KRAS* activating mutations was slightly lower in EOPC than in LOPC samples, but this difference was not statistically significant. However, our set of EOPC samples showed a significantly higher rate of *CDKN2A* mutations than reported in LOPC (*P* = 0.0007) (Table 3A).

**Table 3:** Meta‐synthesis results. Comparison of (A) somatic mutations and (B) protein expression between our data and reported data for EOPC and LOPC (*n* = number of cases (%)).

(A) Somatic mutations							
	LOPC ICGC ≥ 50 years (*n* = 659)	EOPC ICGC < 50 years (*n* = 63)	*P*‐value (LOPC vs. EOPC ICGC)	EOPC our results < 50 years (*n* = 33)	*P*‐value (LOPC ICGC vs. EOPC our results)	Total EOPC (*n* = 96)	*P*‐value (LOPC ICGC vs. total EOPC)
*KRAS*	617 (94%)	57 (90.5%)	ns	29 (88%)	ns	86 (90%)	ns
*CDKN2A*	155 (24%)	11 (17.5%)	ns	17 (52%)	0.0007	28 (29%)	ns
*TP53*	483 (73%)	46 (73%)	ns	27 (82%)	ns	73 (76%)	ns
*SMAD4*	201 (31%)	16 (25.4%)	ns	11 (33%)	ns	27 (28%)	ns

(B) Immunohistochemistry							
	LOPC published reports ≥ 50 years^a^	EOPC published reports < 50 years^b^	*P*‐value (LOPC vs. EOPC)	EOPC our results < 50 years	*P*‐value (LOPC vs. EOPC our results)	Total EOPC	*P*‐value (LOPC vs. total EOPC)
p16 loss	426/612 (69%)	7/7 (100%)	ns	30/35 (86%)	0.055	37/42 (88%)	0.0085
p53 alterations	426/612 (69%)	13/23 (56.5%)	ns	33/35 (94%)	0.0009	46/58 (79%)	ns
SMAD4 loss	282/640 (44%)	8/13 (61.5%)	ns	25/35 (71%)	0.0025	33/47 (70%)	0.0007

^a^ Hua et al. [42], Schlitter et al. [45], Qian et al. [46], Oshima et al. [47].

^b^ Hua et al. [42], Del Chiaro et al. [43], Luttges et al. [4], Bergmann et al. [44].

Immunohistochemistry results for p16, p53 and SMAD4 in our EOPC series were compared with the data from published reports where age of the patients was specified [4, 42, 43, 44, 45, 46, 47]. The results are summarised in Table 3B and Table S6. Loss of p16 was higher than in LOPC and statistically significant only when our data were combined with the published EOPC data (*P* = 0.0085) (Table 3B). A significantly higher rate of p53 alterations (*P* = 0.0009) was seen in our data (Table 3B). The analysis also revealed a significantly higher loss of SMAD4 protein in our EOPC than in LOPC data (*P* = 0.0025), which was consistent between all combined data comparisons (*P* = 0.0007) (Table 3B).

### 3.5 Analysis of intra‐ and intertumoural heterogeneity

**Fig. 3:**
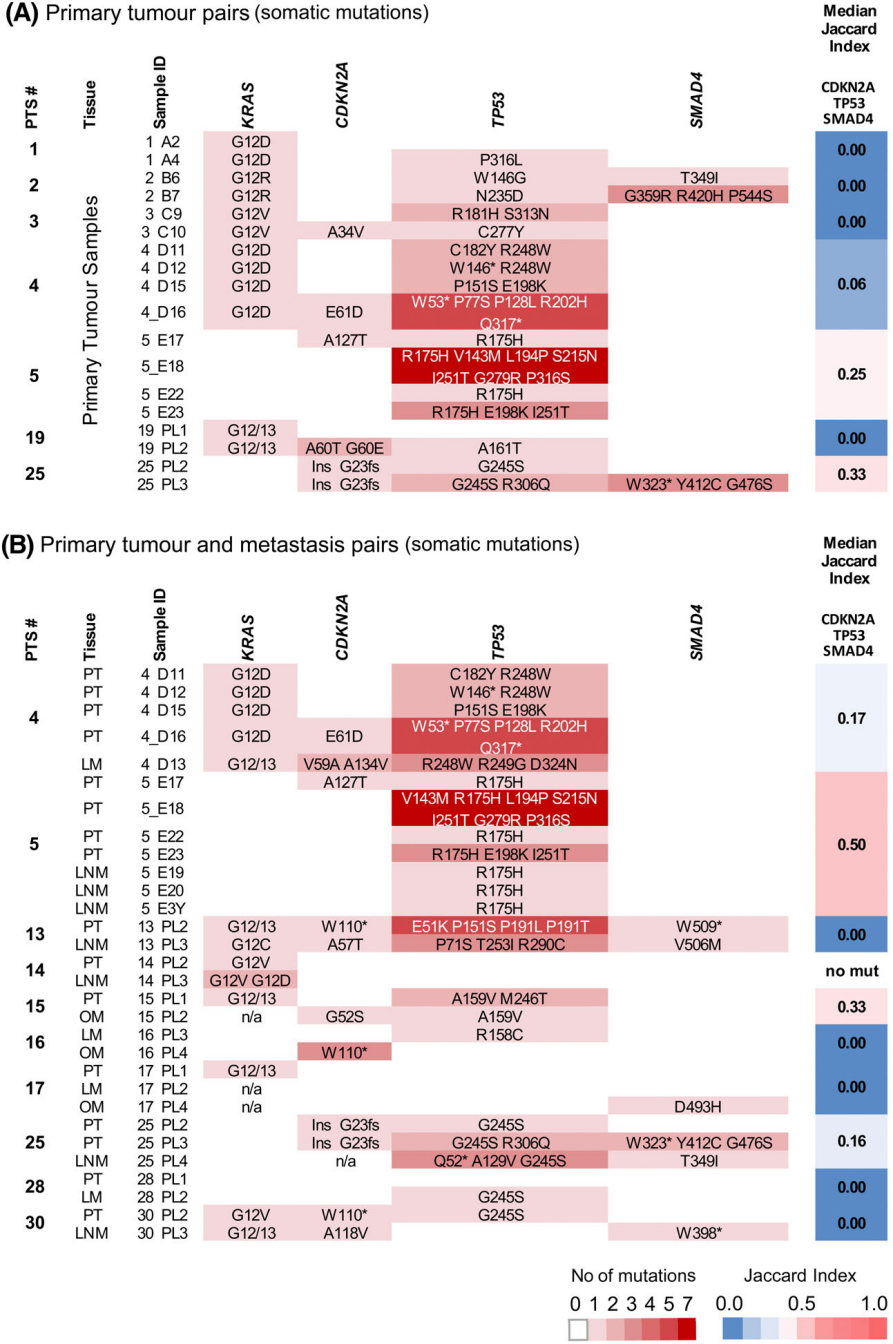
Intratumoural heterogeneity. (A) Primary tumour pairs, somatic mutations; (B) primary tumour‐metastasis pairs, somatic mutations. Each row is a sample, while columns represent genes. The intensity of colour reflects the number of mutations for each sample as indicated in the key. The Median Jaccard Index of similarity is reported for each patient. LM, liver metastasis; LNM, lymph node metastasis; OM, omental metastasis; PT, primary tumour.

Multiple primary tumours were available from seven patients (patients n. 1, n. 2, n. 3, n. 4, n. 5, n. 19 and n. 25). Nine primary tumours had one or more matched metastatic lesions, including lymph nodes (patients n. 5, n. 13, n. 14, n. 25, n. 30), or distant metastases (patients n. 4, n. 15, n. 17, n. 28); patient n. 16 had samples from two distant metastases (Table S1). The intratumoural heterogeneity as quantified by the Jaccard Index of similarities for each primary tumour sample group is shown in Fig. 3A and primary tumour‐metastasis pairs within each patient in Fig. 3B. *KRAS* mutations were shared in primary tumour samples within each patient and between primary tumours and metastatic tissues in all the cases, except for the patient n. 14, who in addition to a G12V in the primary tumour also carried a G12D variant at a higher frequency in the matched lymph node metastasis.

In contrast, a high level of intratumoural heterogeneity was calculated for *CDKN2A*, *SMAD4* and particularly *TP53* in both the number and the type of variants; the Jaccard Index varied from 0 to 0.33 in primary tumour pairs (Fig. 3A) and from 0 to 0.50 in primary tumour‐metastasis pairs (Fig. 3B). Four patients (Patients n. 1, n. 2, n. 3 and n. 19) had no common mutations within their multiple primary tumour tissues, exhibiting thus a Jaccard index = 0 (100% heterogeneity). Patients n. 4, n. 5 and n. 25 also presented high heterogeneity with Jaccard index values of 0.06, 0.25 and 0.33, respectively (Fig. 3A).

The matched primary tumour‐metastases pairs were also characterised by high level of heterogeneity. All the variants in patients n. 13, n. 16, n. 17, n. 28 and n. 30 differed (Jaccard index 0), while patients n. 4, n. 5, n. 15 and n. 25 showed 0.17, 0.50, 0.33 and 0.16, respectively (Fig. 3B).

When samples from different patients were compared, *CDKN2A*, *TP53* and *SMAD4* again showed high heterogeneity (Jaccard index: 0.04, 0.2 and 0.14, respectively) (Fig. S1).

## 4 Discussion

To understand the early onset of PDAC in young patients, we studied one of the largest EOPC series comprising majority of patients of Central/Eastern European origin, which are characteristically more affected by EOPC than patients in other countries [5, 6].

The simplest underlying reason for EOPC could be an increased growth rate [48], which we assessed by immunolabelling of the nuclear antigen Ki67, a commonly utilised marker of proliferation [49]. However, the median of the Ki67 index for our dataset (25%) was similar to that reported for LOPC [37, 50]. Therefore, pancreatic cancer in younger patients appears not to be associated with a more proliferative phenotype.

We next assessed the molecular status of established PDAC drivers, *KRAS*, *CDKN2A*, *TP53* and *SMAD4* [23, 51], and the protein expression of the latter three tumour suppressors.

*KRAS* analysis showed that the majority (88%) of EOPC patients carried common activating mutations, which is somewhat lower than the frequency reported for older PDAC patients (92–96%) [19, 52, 53, 54], and was shown in previous studies on limited number of samples (six [43], seven [44] and 17 [55]). Ben‐Aharon et al. [19] concluded that *KRAS* mutation frequency is significantly lower (83% of *n* = 47 EOPC vs. 96% of *n* = 87 LOPC), but The Cancer Genomic Atlas Research Network (TCGA) data showed *KRAS* alteration in all 20 EOPC patients [54], and both Raffenne et al. [20] (90.5% in *n* = 53 EOPC and 91% in *n* = 89 LOPC) and Tsang et al. [56] (78.8% in *n* = 117 EOPC and 86% in *n* = 165 LOPC) found no significant differences in the rate of *KRAS* mutations between the two age groups. Our meta‐synthesis showed no statistically significant difference between the two age groups. We can conclude that *KRAS* mutations probably play an equally important role in EOPC and LOPC. Overall, although alternative pathways in *KRAS* wild‐type tumours were described [15, 55, 57], these are not age‐associated [54] and so are unlikely to be the specific underlying cause of EOPC.

We found a significantly higher rate of *CDKN2A* somatic mutations (52%) than described previously in LOPC (ICGC meta‐synthesis). At present, the relevance of this finding is uncertain because, at the protein level, loss of p16 expression in our EOPC cohort was comparable to that reported for PDAC in both age groups (commonly between 70% and 98%) [32, 46, 47, 58].

Regarding *TP53* somatic mutations, no significant differences in EOPC and LOPC were seen in both our study and previous studies (ICGC meta‐synthesis). The *TP53* variants found affect mainly the DNA binding site of p53 and were shared by both age groups. However, at the protein level, p53 aberrations affected 33 of the 35 EOPC patients, with a significantly higher rate of nuclear p53 accumulation (*n* = 27 of *n* = 35) than reported for older patients (*P* = 0.0009). Importantly, p53 nuclear accumulation was shown to be a marker of chromosomal instability [59, 60], which is known to result in extensive intratumoural heterogeneity [61]. Furthermore, aberrant p53, when combined with activated *KRAS* ^*G12D*^, leads to accelerated cancer onset [59, 62] and halves the time of cancer progression from pre‐neoplastic lesions to metastatic PDAC in a mouse model [60]. Both of these changes (elevated nuclear p53 accumulation and high intratumoral heterogeneity) were observed in our study and might explain the accelerated development of PDAC in EOPC patients.

The significantly higher loss of SMAD4 protein expression seen in EOPC (*P* = 0.003) is consistent with the finding of previous reports [19, 44]. SMAD4 is a transcription factor and a central effector of TGF‐β signalling pathway, a potent inhibitor of cell growth and survival [63] aberrations of which play a major role in PDAC progression [32, 53]. Mice with combined expression of *Kras* ^*G12D*^ and *Smad4* deletion show accelerated tumour progression and have significantly reduced survival [64]. Of note, SMAD4 deficiency in *Cre LSL‐Kras* ^*G12D*^ *Smad4* ^*lox/lox*^ mice led to a significant increase in acinar‐ductal metaplasia and in both the number and size of early low‐grade PanINs [64], which mimics our finding of a higher number of PanIN lesions in EOPC [22]. Accelerated tumour progression was more pronounced when mutated *Kras* and *Smad4* loss were combined with *Cdkn2a/Arf* loss, which resulted in PDAC in 12/13 mice and IPMN in 1/13, and concomitant PDAC and IPMN in 4 of these 13 animals [64]. Interestingly, two of our EOPC were associated with IPMN, which again mimics the above findings in mice. Our analysis using human samples is of necessity retrospective in nature and therefore provides only a snapshot in time rather than allowing study of temporal progression, which is only available in animal models. We can therefore only speculate about the possibility of earlier involvement of mutated *SMAD4* in the accelerated development of EOPC.

Multiple samples for more than half of EOPC patients enabled us to assess tumour heterogeneity. Interestingly, heterogeneity was not a feature of *KRAS* mutations, which were largely shared between different primary tumours as well as between primary tumours and matched metastases. Minimal *KRAS* heterogeneity was previously described by Hashimoto et al. [65], confirming that metastatic clones are derived from an initiating primary tumour and reinforcing the hypothesis that oncogenic activation of *KRAS* is the earliest PDAC driver [65] in both EOPC and LOPC.

In contrast to *KRAS*, a high level of heterogeneity was observed for *TP53*, *CDKN2A* and *SMAD4*, at both intra‐ and intertumoural levels. The heterogeneity reached up to 100%, with multiple different mutations occurring at a low rate in different samples. Trivedi et al. [66] reported evidence of high heterogeneity in 113 paired patient samples of primary and metastatic PDACs of all ages, while lack of or minimal heterogeneity in *CDKN2A*, *TP53* and *SMAD4* was reported in four LOPC (≥ 59 years) [39]. Minimal heterogeneity was also reported by Reiter et al. [67] and Brar et al. [68]. However, samples in the latter study were not matched, and the age of the patients was not taken into account. While different technical approaches, patient cohorts and number of samples analysed across different studies make direct comparison challenging, as protein alterations were present in both primary and metastatic lesions, our study suggests that the high intra‐ and inter‐tumoural heterogeneity in EOPC patients could play an important role in accelerated development of PDAC in young patients and should thus be further investigated.

Interestingly, in four EOPC patients (n. 16, n. 17, n. 30 and n. 33), a discrepancy in protein loss between the matched primary and metastatic lesions was seen, raising the possibility of subclonality and early metastatic seeding in EOPC patients.

## 5 Conclusions

In summary, the cumulative effect of alterations affecting all four PDAC drivers observed in the majority of EOPC patients, combined with high levels of tumour heterogeneity, could be one of the mechanisms underlying such an early onset of this malignancy in young patients.

## Conflict of interest

The authors declare no conflict of interest.

## Author contributions

SD and TC‐J designed the study. LL, BD, KS, IE and RMF provided samples and helped with data analysis. LL and CN interpreted the IHC data. EC, A‐MB, TG and SMD contributed the material and helped in experimental design. SD, LL and TC‐J wrote the paper. All authors reviewed the manuscript.

### Peer review

The peer review history for this article is available at https://www.webofscience.com/api/gateway/wos/peer‐review/10.1002/1878‐0261.13576.

## Supporting information


**Fig. S1.** Intertumoural heterogeneity.

**Table S1.** Sample and analysis details.

**Table S2.** Amplicon details and primer sequences.

**Table S3.** Sequence analysis results.

**Table S4.** *KRAS* mutation results.

**Table S5.** Immunohistochemistry results.

**Table S6.** Reference studies for IHC meta‐synthesis.

## Data Availability

All relevant data are provided within the manuscript and the Supporting Information files and are available without restriction.
