# Supplementary material for: Molecular characteristics of early‐onset pancreatic ductal adenocarcinoma
Source: Mol Oncol. 2024 Jan 3;18(3):677–90. doi: 10.1002/1878-0261.13576 (PMC10920080; doi:10.1002/1878-0261.13576)
Supplement: Supplementary file 1 — Fig. S1. Intertumoural heterogeneity. [file MOL2-18-677-s005.docx]

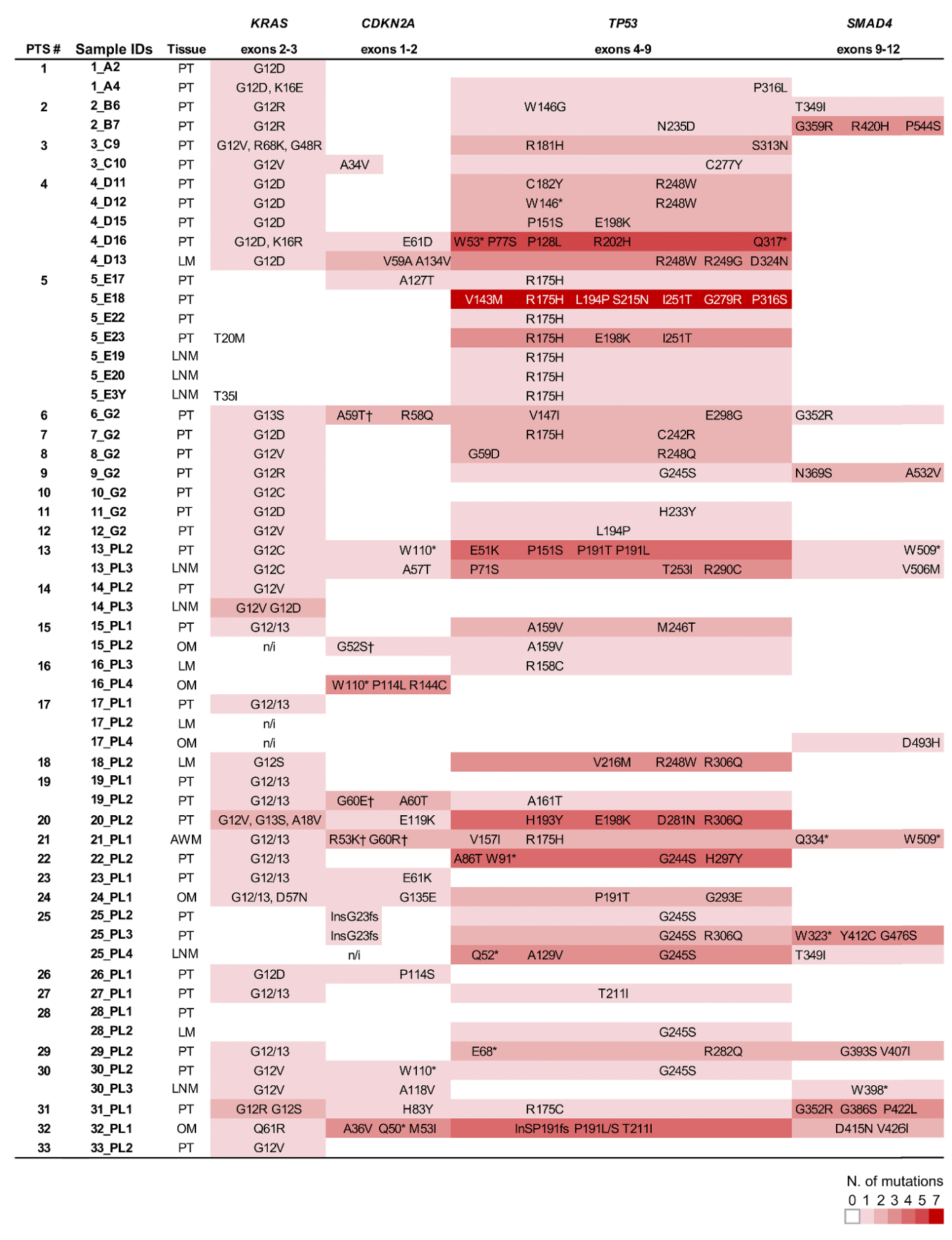
**Figure S1.** **Intertumoral heterogeneity.** All the mutations of *KRAS*, *CDKN2A*, *TP53* and *SMAD4* for all the samples are reported in the figure. Each row is a sample, while columns represent genes. Colour intensity is proportional to the number of mutations as indicated at the bottom of the table. AWM, abdominal wall metastasis; LM, liver metastasis; LNM, lymph node metastasis; n/i, not interpretable; OM, omental metastasis; PT, Primary Tumour.
