# Supplementary material for: Molecular characteristics of early‐onset pancreatic ductal adenocarcinoma
Source: Mol Oncol. 2024 Jan 3;18(3):677–90. doi: 10.1002/1878-0261.13576 (PMC10920080; doi:10.1002/1878-0261.13576)
Supplement: Supplementary file 2 — Table S1. Sample and analysis details. [file MOL2-18-677-s004.docx]

**Table S1.** Sample details and analyses performed.

| **Patient ID #** | **Origin** | **Age** | **Gender** | **Stage** | **Sample ID** | **Tissue type** | **Analyses performed** | | |
| --- | --- | --- | --- | --- | --- | --- | --- | --- | --- |
| **1** | UK | 43 | M | IIB | 1_A1 | N Pancreas | NGS |  |  |
|  |  |  |  |  | 1_A2 | Primary Tu | NGS | IHC | ddPCR |
|  |  |  |  |  | 1_A3 | Primary Tu |  | IHC |  |
|  |  |  |  |  | 1_A4 | Primary Tu | NGS |  |  |
| **2** | UK | 44 | M | IIB | 2_B5 | N Duodenum | NGS |  |  |
|  |  |  |  |  | 2_B6 | Primary Tu | NGS |  |  |
|  |  |  |  |  | 2_B7 | Primary Tu | NGS | IHC | ddPCR |
| **3** | UK | 49 | M | IIB | 3_C8 | N Pancreas | NGS |  |  |
|  |  |  |  |  | 3_C9 | Primary Tu | NGS | IHC |  |
|  |  |  |  |  | 3_C10 | Primary Tu | NGS | IHC | ddPCR |
| **4** | Croatia | 45 | M | IV | 4_D14 | N Pancreas | NGS | IHC |  |
|  |  |  |  |  | 4_D11 | Primary Tu | NGS | IHC | ddPCR |
|  |  |  |  |  | 4_D12 | Primary Tu | NGS | IHC |  |
|  |  |  |  |  | 4_D15 | Primary Tu | NGS | IHC |  |
|  |  |  |  |  | 4_D16 | Primary Tu | NGS | IHC |  |
|  |  |  |  |  | 4_D13 | Liver met | NGS | IHC | ddPCR |
| **5** | Croatia | 42 | F | n/a | 5_E21 | N Pancreas | NGS |  | ddPCR |
|  |  |  |  |  | 5_E17 | Primary Tu | NGS | IHC |  |
|  |  |  |  |  | 5_E18 | Primary Tu | NGS | IHC | ddPCR |
|  |  |  |  |  | 5_E22 | Primary Tu | NGS | IHC |  |
|  |  |  |  |  | 5_E23 | Primary Tu | NGS | IHC | ddPCR |
|  |  |  |  |  | 5_E19 | LN met | NGS | IHC |  |
|  |  |  |  |  | 5_E20 | LN met | NGS | IHC |  |
|  |  |  |  |  | 5_E3Y | LN met | NGS | IHC |  |
| **6** | Germany | 31 | M | III | 6_G1 | N Pancreas | NGS |  |  |
|  |  |  |  |  | 6_G2 | Primary Tu | NGS |  |  |
| **7** | Germany | 46 | F | II | 7_G1 | N Pancreas | NGS |  |  |
|  |  |  |  |  | 7_G2 | Primary Tu | NGS | IHC |  |
| **8** | Germany | 42 | M | IV | 8_G1 | N Pancreas | NGS |  |  |
|  |  |  |  |  | 8_G2 | Primary Tu | NGS | IHC | ddPCR |
| **9** | Germany | 34 | F | IV | 9_G1 | N Pancreas | NGS |  |  |
|  |  |  |  |  | 9_G2 | Primary Tu | NGS | IHC | ddPCR |
| **10** | Germany | 32 | M | III | 10_G1 | N Pancreas | NGS |  |  |
|  |  |  |  |  | 10_G2 | Primary Tu | NGS | IHC | ddPCR |
| **11 *** | Germany | 35 | M | III | 11_G1 | N Pancreas | NGS |  |  |
|  |  |  |  |  | 11_G2 | Primary Tu | NGS | IHC | ddPCR |
| **12** | Germany | 39 | F | III | 12_G1 | N Pancreas | NGS |  |  |
|  |  |  |  |  | 12_G2 | Primary Tu | NGS |  |  |
| **13** | Poland | 49 | F | IIB | 13_PL1 | N Pancreas | NGS | IHC |  |
|  |  |  |  |  | 13_PL2 | Primary Tu | NGS | IHC | ddPCR |
|  |  |  |  |  | 13_PL3 | LN met | NGS | IHC | ddPCR |
| **14** | Poland | 43 | F | III | 14_PL1 | N Pancreas | NGS | IHC |  |
|  |  |  |  |  | 14_PL2 | Primary Tu | NGS | IHC |  |
|  |  |  |  |  | 14_PL3 | LN met | NGS | IHC |  |
| **15** | Poland | 47 | F | IV | 15_PL1 | Primary Tu | NGS | IHC | ddPCR |
|  |  |  |  |  | 15_PL2 | Om met | NGS | IHC |  |
| **16** | Poland | 39 | M | IV | 16_PL1 | N Liver | NGS | IHC |  |
|  |  |  |  |  | 16_PL2 | Primary Tu |  | IHC | ddPCR |
|  |  |  |  |  | 16_PL3 | Liver met | NGS | IHC | ddPCR |
|  |  |  |  |  | 16_PL4 | Om met | NGS | IHC | ddPCR |
| **17** | Poland | 32 | F | IV | 17_PL1 | Primary Tu | NGS | IHC | ddPCR |
|  |  |  |  |  | 17_PL2 | Liver met | NGS | IHC |  |
|  |  |  |  |  | 17_PL3 | LN met |  | IHC |  |
|  | Poland |  | F |  | 17_PL4 | Om met | NGS | IHC |  |
|  |  |  |  |  | 17_PL5 | AW met |  | IHC |  |
| **18** | Poland | 48 | M | IV | 18_PL1 | N Liver | NGS | IHC |  |
|  |  |  |  |  | 18_PL2 | Liver met | NGS | IHC |  |
| **19** | Poland | 43 | M | III | 19_PL1 | Primary Tu | NGS | IHC | ddPCR |
|  |  |  |  |  | 19_PL2 | Primary Tu | NGS | IHC | ddPCR |
| **20** | Poland | 44 | M | IA | 20_PL1 | N Pancreas | NGS | IHC |  |
|  |  |  |  |  | 20_PL2 | Primary Tu | NGS | IHC |  |
| **21** | Poland | 48 | M | IV | 21_PL1 | AW met | NGS | IHC | ddPCR |
|  |  |  |  |  | 21_PL2 | Per met |  | IHC |  |
| **22 *** | Poland | 42 | F | IB | 22_PL1 | N Duodenum | NGS | IHC |  |
|  |  |  |  |  | 22_PL2 | Primary Tu | NGS | IHC | ddPCR |
| **23** | Poland | 49 | F | IV | 23_PL1 | Primary Tu | NGS | IHC | ddPCR |
|  |  |  |  |  | 23_PL2 | Liver met |  | IHC |  |
| **24** | Poland | 48 | M | IV | 24_PL1 | Om met | NGS | IHC | ddPCR |
| **25 ^** | Poland | 43 | F | IIB | 25_PL1 | N Pancreas | NGS | IHC |  |
|  |  |  |  |  | 25_PL2 | Primary Tu | NGS | IHC | ddPCR |
|  |  |  |  |  | 25_PL3 | Primary Tu | NGS | IHC |  |
|  |  |  |  |  | 25_PL4 | LN met | NGS | IHC |  |
| **26** | Poland | 38 | M | IV | 26_PL1 | Primary Tu | NGS | IHC | ddPCR |
| **27** | Poland | 48 | F | III | 27_PL1 | Primary Tu | NGS | IHC | ddPCR |
| **28** | Poland | 49 | F | IV | 28_PL1 | Primary Tu | NGS | IHC | ddPCR |
|  |  |  |  |  | 28_PL2 | Liver met | NGS | IHC |  |
| **29** | Poland | 49 | F | III | 29_PL1 | N Duodenum | NGS | IHC |  |
|  |  |  |  |  | 29_PL2 | Primary Tu | NGS | IHC | ddPCR |
| **30 ^** | Poland | 49 | M | III | 30_PL1 | N Pancreas | NGS | IHC |  |
|  |  |  |  |  | 30_PL2 | Primary Tu | NGS | IHC |  |
|  |  |  |  |  | 30_PL3 | LN met | NGS | IHC | ddPCR |
| **31** | Poland | 43 | F |  | 31_PL1 | Primary Tu | NGS | IHC |  |
| **32** | Poland | 48 | F | IV | 32_PL1 | Om met | NGS | IHC |  |
| **33** | Poland | 49 | M | III | 33_PL1 | N Duodenum | NGS | IHC |  |
|  |  |  |  |  | 33_PL2 | Primary Tu | NGS | IHC |  |
|  |  |  |  |  | 33_PL3 | LN met |  | IHC |  |
| **34** | Poland | 44 | F | IV | 34_PL1 | Om met |  | IHC |  |
| **35** | Poland | 48 | M |  | 35_PL1 | LN met |  | IHC |  |
| **36** | Poland | 44 | F | IV | 36_PL1 | Per met |  | IHC |  |
| **37** | Poland | 33 | F | IV | 37_PL1 | Om met |  | IHC |  |

*PDAC associated with Intraductal Papillary Mucinous Neoplasm (IPMN); ^Adenosquamous carcinoma; AW, abdominal wall; ddPCR. droplet digital PCR; F, female; IHC, immunohistochemistry; LN, lymph node; M, male; met, metastasis; N, normal; NGS, next generation sequencing; Om, omental; Per, peritoneum; Tu, tumour.
