# Supplementary material for: Molecular characteristics of early‐onset pancreatic ductal adenocarcinoma
Source: Mol Oncol. 2024 Jan 3;18(3):677–90. doi: 10.1002/1878-0261.13576 (PMC10920080; doi:10.1002/1878-0261.13576)
Supplement: Supplementary file 3 — Table S2. Amplicon details and primer sequences. [file MOL2-18-677-s007.docx]

**Table S2.** Amplicon details and primer sequences.

| **Gene** | **Exon** | **UCSC GRCh37/ hg19 Start** | **UCSC GRCh37/ hg19 End** | **Forward Primer** | **Reverse Primer** | **Product Length** | **GC %** |
| --- | --- | --- | --- | --- | --- | --- | --- |
| KRAS | 2 | 25398147 | 25398331 | ACACTGACGACATGGTTCTACACCTTTATCTGTATCAAAGAATGGTCCT | TACGGTAGCAGAGACTTGGTCTAGGCCTGCTGAAAATGACTGAAT | 185 | 39 |
| KRAS | 3 | 25380209 | 25380388 | ACACTGACGACATGGTTCTACAGGCAAATACACAAAGAAAGCCCTC | TACGGTAGCAGAGACTTGGTCTAGGTGCACTGTAATAATCCAGAC | 180 | 47 |
| TP53 | 4 | 7579261 | 7579460 | ACACTGACGACATGGTTCTACATACGGCCAGGCATTGAAGTC | TACGGTAGCAGAGACTTGGTCTCACCAGCAGCTCCTACACC | 200 | 61 |
| TP53 | 4 | 7579385 | 7579580 | ACACTGACGACATGGTTCTACATTCTGGGAAGGGACAGAAGATGA | TACGGTAGCAGAGACTTGGTCTCGTCCCAAGCAATGGATGATTTG | 196 | 61 |
| TP53 | 4 | 7579442 | 7579625 | ACACTGACGACATGGTTCTACAGGTGTAGGAGCTGCTGGTG | TACGGTAGCAGAGACTTGGTCTCTGGTCCTCTGACTGCTCTTTTC | 184 | 57 |
| TP53 | 5 | 7578320 | 7578505 | ACACTGACGACATGGTTCTACATGGGGACCCTGGGCAA | TACGGTAGCAGAGACTTGGTCTCTGTGCAGCTGTGGGTTGATT | 186 | 66 |
| TP53 | 5 | 7578408 | 7578598 | ACACTGACGACATGGTTCTACACCTCACAACCTCCGTCATGTG | TACGGTAGCAGAGACTTGGTCTCTTGTGCCCTGACTTTCAACTCT | 191 | 59 |
| TP53 | 6 | 7578123 | 7578317 | ACACTGACGACATGGTTCTACACACTGACAACCACCCTTAACCC | TACGGTAGCAGAGACTTGGTCTCCTCTGATTCCTCACTGATTGCT | 195 | 52 |
| TP53 | 7 | 7577471 | 7577663 | ACACTGACGACATGGTTCTACAGTGTGCAGGGTGGCAAGT | TACGGTAGCAGAGACTTGGTCTCCACAGGTCTCCCCAAGG | 193 | 57 |
| TP53 | 8 | 7576940 | 7577119 | ACACTGACGACATGGTTCTACAAAGGAAAGGTGATAAAAGTGAATCTG | TACGGTAGCAGAGACTTGGTCTTGTTTGTGCCTGTCCTGGG | 180 | 56 |
| TP53 | 8 | 7576999 | 7577194 | ACACTGACGACATGGTTCTACATTCTTGTCCTGCTTGCTTACCTC | TACGGTAGCAGAGACTTGGTCTCCTTACTGCCTCTTGCTTCTCTT | 196 | 55 |
| TP53 | 9 | 7576755 | 7576954 | ACACTGACGACATGGTTCTACAAATGCCCCAATTGCAGGTAAAAC | TACGGTAGCAGAGACTTGGTCTTTATCACCTTTCCTTGCCTCTTT | 200 | 44 |
| CDKN2A | 1 | 21974630 | 21974809 | ACACTGACGACATGGTTCTACACAGAGTCGCCCGCCAT | TACGGTAGCAGAGACTTGGTCTGAGCAGCATGGAGCCTTCG | 180 | 72 |
| CDKN2A | 1 | 21974775 | 21974974 | ACACTGACGACATGGTTCTACATGGCCAGCCAGTCAGC | TACGGTAGCAGAGACTTGGTCTTTTGAGGGACAGGGTCGGAG | 200 | 73 |
| CDKN2A | 2 | 21970819 | 21971012 | ACACTGACGACATGGTTCTACAGCTGAACTTTCTGTGCTGGAAAA | TACGGTAGCAGAGACTTGGTCTGACCTGGCTGAGGAGCTG | 194 | 59 |
| CDKN2A | 2 | 21970907 | 21971087 | ACACTGACGACATGGTTCTACAGACCTTCCGCGGCATCTAT | TACGGTAGCAGAGACTTGGTCTCTGGACACGCTGGTGGTG | 181 | 72 |
| CDKN2A | 2 | 21970997 | 21971176 | ACACTGACGACATGGTTCTACAGCTCCTCAGCCAGGTCCA | TACGGTAGCAGAGACTTGGTCTAGCTGCTGCTGCTCCAC | 180 | 75 |
| CDKN2A | 2 | 21971134 | 21971326 | ACACTGACGACATGGTTCTACAGGGTCGGCGCAGTTGG | TACGGTAGCAGAGACTTGGTCTTAATTAGACACCTGGGGCTTGTG | 193 | 64 |
| CDKN2A | 3 | 21968520 | 21968703 | ACACTGACGACATGGTTCTACACAGGGTTGCAAGAAGAAAACGAG | TACGGTAGCAGAGACTTGGTCTGAGGAGCGCCAGAGCC | 184 | 57 |
| CDKN2A | 3 | 21968618 | 21968816 | ACACTGACGACATGGTTCTACACATCCCCAGGCATCTTTTGCAC | TACGGTAGCAGAGACTTGGTCTGCAGAAGCCAGAGCACATGAATA | 199 | 54 |
| SMAD4 | 9 | 48591710 | 48591889 | ACACTGACGACATGGTTCTACAAGGAAAAACTGTGTTGTGGAGTG | TACGGTAGCAGAGACTTGGTCTTCAACAGTAACAATAGGGCAGCTT | 180 | 40 |
| SMAD4 | 9 | 48591795 | 48591979 | ACACTGACGACATGGTTCTACACCTGAGTATTGGTGTTCCATTGC | TACGGTAGCAGAGACTTGGTCTTACCTTGCTCTCTCAATGGCTTC | 185 | 45 |
| SMAD4 | 9 | 48591867 | 48592062 | ACACTGACGACATGGTTCTACAAGCTGCCCTATTGTTACTGTTGA | TACGGTAGCAGAGACTTGGTCTTGTACATGGGAAAACATAACCTTGA | 196 | 40 |
| SMAD4 | 10 | 48593261 | 48593460 | ACACTGACGACATGGTTCTACAAGCTATCTTTTGGTTTTATGTGATCTTT | TACGGTAGCAGAGACTTGGTCTTCACTAAGGCACCTGACCC | 200 | 30 |
| SMAD4 | 10 | 48593379 | 48593563 | ACACTGACGACATGGTTCTACATCTTCCTAAGGTTGCACATAGGC | TACGGTAGCAGAGACTTGGTCTACTAACCTTTATATATGCACTTGGGT | 185 | 46 |
| SMAD4 | 10 | 48593436 | 48593635 | ACACTGACGACATGGTTCTACAATGTTTGGGTCAGGTGCCTTAGT | TACGGTAGCAGAGACTTGGTCTCCTTCCACCCAGATTTCAATTCTTTT | 200 | 40 |
| SMAD4 | 11 | 48602942 | 48603138 | ACACTGACGACATGGTTCTACACATTGGTTTTTAATGTATGGAATTTTTCTTTATG | TACGGTAGCAGAGACTTGGTCTGGAGCTATTCCACCTACTGATCC | 197 | 47 |
| SMAD4 | 11 | 48603090 | 48603278 | ACACTGACGACATGGTTCTACACCGTGGCAGGAAACATCC | TACGGTAGCAGAGACTTGGTCTTCAAAAATGTCATCATCCCAGTAAAA | 189 | 34 |
| SMAD4 | 12 | 48604578 | 48604763 | ACACTGACGACATGGTTCTACAGAAGAGATCACCCTGTCCCTCT | TACGGTAGCAGAGACTTGGTCTAGTGAATTTCAATCCAGCAAGGTG | 186 | 46 |
| SMAD4 | 12 | 48604680 | 48604863 | ACACTGACGACATGGTTCTACATCAGGATGAGTTTTGTGAAAGGC | TACGGTAGCAGAGACTTGGTCTGTTAAGGGCCCCAACGGTAAA | 184 | 49 |
