# Supplementary material for: Molecular characteristics of early‐onset pancreatic ductal adenocarcinoma
Source: Mol Oncol. 2024 Jan 3;18(3):677–90. doi: 10.1002/1878-0261.13576 (PMC10920080; doi:10.1002/1878-0261.13576)
Supplement: Supplementary file 4 — Table S3. Sequence analysis results. [file MOL2-18-677-s001.docx]

**Table S3.** Mutation analysis results. The list of amino acid substitutions is reported for each patient.

|  |  |  | **KRAS** | **CDKN2A** | | **TP53** | | **SMAD4** |
| --- | --- | --- | --- | --- | --- | --- | --- | --- |
| **Pts #** | **Sample ID** | **Tissue** | **Exons 2-3** | **Exon 1 (p12)** | **Exons 1-2** | **Exons 4-9** | | **Exons 9-10, 12** |
| **1** | 1_A2 | PT | G12D K16E |  |  |  |  |  |
|  | 1_A4 | PT | G12D |  |  | P316L | |  |
| **2** | 2_B6 | PT | G12R |  |  | W146G | | T349I |
|  | 2_B7 | PT | G12R |  |  | N235D | | G359R  R420H P544S |
| **3** | 3_C9 | PT | G12V G48R R68K |  |  | R181H | S313N |  |
|  | 3_C10 | PT | G12V |  | A34V | C277Y | |  |
| **4** | 4_D11 | PT | G12D |  |  | C182Y | R248W |  |
|  | 4_D12 | PT | G12D |  |  | W146* | R248W |  |
|  | 4_D15 | PT | G12D |  |  | P151S | E198K |  |
|  | 4_D16 | PT | G12D K16R |  | E61D | W53* P77S P128L R202H Q317* | |  |
|  | 4_D13 | LM | G12/13 |  | V59A A134V | R248WR249G D324N | |  |
| **5** | 5_E17 | PT |  |  | A127T | R175H |  |  |
|  | 5_E18 | PT |  |  |  | R175H V143M S215N L194P I251T G279R P316S | |  |
|  | 5_E22 | PT |  |  |  | R175H |  |  |
|  | 5_E23 | PT | T20M |  |  | R175H | E198K I251T |  |
|  | 5_E19 | LNM |  |  |  | R175H |  |  |
|  | 5_E20 | LNM |  |  |  | R175H |  |  |
|  | 5_E3Y | LNM | T35I |  |  | R175H |  |  |
| **6** | 6_G2 | PT | G13S | A59T | R58Q | V147I | E298G | G352R |
| **7** | 7_G2 | PT | G12D |  |  | R175H | C242R |  |
| **8** | 8_G2 | PT | G12V |  |  | G59D | R248Q |  |
| **9** | 9_G2 | PT | G12R |  |  | G245S | | N369S A532V |
| **10** | 10_G2 | PT | G12C |  |  |  |  |  |
| **11** | 11_G2 | PT | G12D |  |  | H233Y | |  |
| **12** | 12_G2 | PT | G12V |  |  | L194P | |  |
| **13** | 13_PL2 | PT | G12/13 |  | W110* | E51K P151S | P191L P191T | W509* |
|  | 13_PL3 | LNM | G12C |  | A57T | P71S | T253I R290C | V506M |
| **14** | 14_PL2 | PT | G12V |  |  |  |  |  |
|  | 14_PL3 | LNM | G12V G12D |  |  |  |  |  |
| **15** | 15_PL1 | PT | G12/13 |  |  | A159V | M246T |  |
|  | 15_PL2 | OM | N/I | G52S |  | A159V |  |  |
| **16** | 16_PL2 | PT |  |  | N/A |  | N/A | N/A |
|  | 16_PL3 | LM |  |  |  | R158C |  |  |
|  | 16_PL4 | OM |  |  | W110* P114L R144C |  |  |  |
| **17** | 17_PL1 | PT | G12/13 |  |  |  |  |  |
|  | 17_PL2 | LM |  |  |  |  |  |  |
|  | 17_PL4 | OM |  |  | P41L |  |  | D493H |
| **18** | 18_PL2 | LM | G12S |  |  | V216M R248W R306Q | |  |
| **19** | 19_PL1 | PT | G12/13 |  |  |  |  |  |
|  | 19_PL2 | PT | G12/13 | G60E | A60T | A161T | |  |
| **20** | 20_PL2 | PT | G12V G13S A18V |  | E119K | H193Y E198K R306Q D281N | |  |
| **21** | 21_PL1 | AWM | G12/13 | R53K G60R |  | R175H V157I | | Q334* W509* |
| **22** | 22_PL2 | PT | G12/13 |  |  | A86T W91* | G244S H297Y |  |
| **23** | 23_PL1 | PT | G12/13 |  | E61K |  |  |  |
| **24** | 24_PL1 | OM | G12/13 |  | G135E | P191T G293E | |  |
| **25** | 25_PL2 | PT |  |  | Ins(1nt)G23fs | G245S | |  |
|  | 25_PL3 | PT |  |  | Ins(1nt)G23fs | G245S R306Q | | W323* Y412C G476S |
|  | 25_PL4 | LNM |  |  |  | Q52* A129V | G245S | T349I |
| **26** | 26_PL1 | PT | G12D |  | P114S |  |  |  |
| **27** | 27_PL1 | PT | G12/13 |  |  | T211I | |  |
| **28** | 28_PL1 | PT |  |  |  |  | |  |
|  | 28_PL2 | LM |  |  |  | G245S | |  |
| **29** | 29_PL2 | PT | G12/13 |  |  | E68* | R282Q | G393S V407I |
| **30** | 30_PL2 | PT | G12V |  | W110* | G245S | |  |
|  | 30_PL3 | LNM | G12/13 |  | A118V |  |  | W398* |
| **31** | 31_PL1 | PT | G12R G12S |  | H83Y | R175C | | G352R G386S P422L |
| **32** | 32_PL1 | OM | Q61R |  | A36V Q50* M53I | Ins_P191fs P191L P191S T211I | | D415N V426I |
| **33** | 33_PL2 | PT | G12V |  |  |  |  |  |

AWM, abdominal wall metastasis; fs, frameshift; Ins, insertion; LM, liver metastasis; LNM, lymph node metastasis; N/A, not available; N/I, not interpretable; OM, omental metastasis; PT, Primary Tumour; PTS, patients; *, stop codon.
