# Supplementary material for: Molecular characteristics of early‐onset pancreatic ductal adenocarcinoma
Source: Mol Oncol. 2024 Jan 3;18(3):677–90. doi: 10.1002/1878-0261.13576 (PMC10920080; doi:10.1002/1878-0261.13576)
Supplement: Supplementary file 5 — Table S4. KRAS mutation results. [file MOL2-18-677-s006.docx]

**Table S4.** *KRAS* mutation results.

|  |  |  | **NGS results** | | **ddPCR results** | |
| --- | --- | --- | --- | --- | --- | --- |
| **Patient ID #** | **Sample ID** | **Tissue Type** | **aa change** | **Allele Frequency** | **ddPCR KRAS mutation** | **Fraction mutated copies** |
| **1** | 1_A2 | Primary Tu | G12D | 12.5% | G12/13 | 19.2% |
|  | 1_A4 | Primary Tu | G12D, K16E | 21.5%, 30.6% |  |  |
| **2** | 2_B6 | Primary Tu | G12R | 75.9% |  |  |
|  | 2_B7 | Primary Tu | G12R | 55.1% | G12/13 | 42.3% |
| **3** | 3_C9 | Primary Tu | G12V, R68K, G48R | 65.8%, 25.8%, 26.6% |  |  |
|  | 3_C10 | Primary Tu | G12V | 100% | G12/13 | 23.3% |
| **4** | 4_D11 | Primary Tu | G12D | 23.8% | G12/13 | 26.5% |
|  | 4_D12 | Primary Tu | G12D | 15.3% |  |  |
|  | 4_D15 | Primary Tu | G12D | 13.6% |  |  |
|  | 4_D16 | Primary Tu | G12D, K16R | 14.2%, 20% |  |  |
|  | 4_D13 | Liver met | No |  | G12/13 | 12.7% |
| **5** | 5_E17 | Primary Tu | No |  |  |  |
|  | 5_E18 | Primary Tu | No |  | No |  |
|  | 5_E22 | Primary Tu | No |  |  |  |
|  | 5_E23 | Primary Tu | T20M | 21% | No |  |
|  | 5_E19 | LN met | No |  |  |  |
|  | 5_E20 | LN met | No |  |  |  |
|  | 5_E3Y | LN met | T35I | 99% |  |  |
| **6** | 6_G2 | Primary Tu | G13S | 17.1% |  |  |
| **7** | 7_G2 | Primary Tu | G12D | 10.6% |  |  |
| **8** | 8_G2 | Primary Tu | G12V | 99.0% | G12/13 | 19.7% |
| **9** | 9_G2 | Primary Tu | G12R |  | G12/13 | 26.6% |
| **10** | 10_G2 | Primary Tu | G12C |  | G12/13 | 10.4% |
| **11** | 11_G2 | Primary Tu | G12D |  | G12/13 | 10.3% |
| **12** | 12_G2 | Primary Tu | G12V | 18.9% |  |  |
| **13** | 13_PL2 | Primary Tu | No |  | G12/13 | 19.7% |
|  | 13_PL3 | LN met | G12C | 36.1% | G12/13 | 1.9% |
| **14** | 14_PL2 | Primary Tu | G12V | 38.7% |  |  |
|  | 14_PL3 | LN met | G12D, G12V | 15.6%, 5.4% |  |  |
| **15** | 15_PL1 | Primary Tu | No |  | G12/13 | 44.0% |
|  | 15_PL2 | Om met | n/i |  |  |  |
| **16** | 16_PL2 | Primary Tu |  |  | No |  |
|  | 16_PL3 | Liver met | No |  | No |  |
|  | 16_PL4 | Om met | n/i |  | No |  |
| **17** | 17_PL1 | Primary Tu | n/i |  | G12/13 | 42.7% |
|  | 17_PL2 | Liver met | No |  |  |  |
|  | 17_PL4 | Om met | n/i |  |  |  |
| **18** | 18_PL2 | Liver met | G12S | 7.3% |  |  |
| **19** | 19_PL1 | Primary Tu | n/i |  | G12/13 | 9.50% |
|  | 19_PL2 | Primary Tu | No |  | G12/13 | 7% |
| **20** | 20_PL2 | Primary Tu | G12V, G13S, A18V | 34.8%, 18.4%, 25% |  |  |
| **21** | 21_PL1 | AW met | No |  | G12/13 | 61.0% |
| **22** | 22_PL2 | Primary Tu | n/i |  | G12/13 | 7.9% |
| **23** | 23_PL1 | Primary Tu | No |  | G12/13 | 35.0% |
| **24** | 24_PL1 | Om met | D57N | 25% | G12/13 | 4.9% |
| **25** | 25_PL2 | Primary Tu | No |  | No |  |
|  | 25_PL3 | Primary Tu | No |  |  |  |
|  | 25_PL4 | LN met | n/i |  |  |  |
| **26** | 26_PL1 | Primary Tu | G12D | 10.4% | G12/13 | 12.3% |
| **27** | 27_PL1 | Primary Tu | n/i |  | G12/13 | 12.5% |
| **28** | 28_PL1 | Primary Tu | n/i |  | No |  |
|  | 28_PL2 | Liver met | n/i |  |  |  |
| **29** | 29_PL2 | Primary Tu | No |  | G12/13 | 22.4% |
| **30** | 30_PL2 | Primary Tu | G12V | 31.2% |  |  |
|  | 30_PL3 | LN met | n/i |  | G12/13 | 2.2% |
| **31** | 31_PL1 | Primary Tu | G12R, G12S | 8.2%, 4% |  |  |
| **32** | 32_PL1 | Om met | Q61R | 15.9% |  |  |
| **33** | 33_PL2 | Primary Tu | G12V | 23.7% |  |  |

AW, abdominal wall; LN, lymph node; met, metastasis; n/i, not interpretable; No, not mutated; Om, omental; Tu, tumour.
