# Supplementary material for: Molecular characteristics of early‐onset pancreatic ductal adenocarcinoma
Source: Mol Oncol. 2024 Jan 3;18(3):677–90. doi: 10.1002/1878-0261.13576 (PMC10920080; doi:10.1002/1878-0261.13576)
Supplement: Supplementary file 6 — Table S5. Immunohistochemistry results. [file MOL2-18-677-s002.docx]

**Table S5.** Immunohistochemistry results.

| **ID #** | **Patient ID** | **Tissue type** | **p16** | **p53**  **(% of cells)** | **SMAD4** | **Ki67** |
| --- | --- | --- | --- | --- | --- | --- |
| **1** | 1_A2 | Primary Tu | Lost | Lost | Lost | n/a |
|  | 1_A3 | Primary Tu | Lost | Lost | Lost | n/a |
| **2** | 2_B7 | Primary Tu | Lost | Overexp (>30%) | Lost | n/a |
| **3** | 3_C9 | Primary Tu | Lost | Lost | Lost | n/a |
|  | 3_C10 | Primary Tu | Lost | Lost | Lost | n/a |
| **4** | 4_D11 | Primary Tu | Lost | Overexp (~30%) | Retained | n/i |
|  | 4_D12 | Primary Tu | Lost | Overexp (~70%) | Retained | 26.5% |
|  | 4_D15 | Primary Tu | Lost | Overexp (~30%) | Retained | 53.6% |
|  | 4_D16 | Primary Tu | Lost | Overexp (~80%) | Retained | 61.0% |
|  | 4_D13 | Liver met | Lost | Overexp (~80%) | Uncertain | 32.0% |
| **5** | 5_E17 | Primary Tu | Lost | Overexp (~60%) | Lost 50% | n/i |
|  | 5_E18 | Primary Tu | Lost | Overexp (~50%) | Retained | 73.3% |
|  | 5_E22 | Primary Tu | Lost | Overexp (~50%) | Retained | n/i |
|  | 5_E23 | Primary Tu | Lost | Overexp (~80%) | Retained | n/i |
|  | 5_E19 | LN met | Lost | Overexp (~70%) | Retained | 15.1% |
|  | 5_E20 | LN met | Lost | Overexp (~40%) | Retained | n/i |
|  | 5_E3Y | LN met | Lost | Overexp (~90%) | Lost 50% | n/i |
| **7** | 7_G2 | Primary Tu | Lost | Overexp (>60%) | Retained | n/a |
| **8** | 8_G2 | Primary Tu | Lost | Overexp (>90%) | Lost | n/a |
| **9** | 9_G2 | Primary Tu | Lost | Overexp (>70%) | Retained | n/a |
| **10** | 10_G2 | Primary Tu | Lost | Overexp (>30%) | Lost | n/a |
| **11** | 11_G2 | Primary Tu | Lost | Overexp (>80%) | Retained | n/a |
| **13** | 13_PL2 | Primary Tu | Lost | Lost | Lost | 8.9% |
|  | 13_PL3 | LN met | Lost | Lost | Lost | 10.4% |
| **14** | 14_PL2 | Primary Tu | Lost | Lost | Lost | 24.5% |
|  | 14_PL3 | LN met | Lost | Lost | Lost | 23.4% |
| **15** | 15_PL1 | Primary Tu | Lost | Overexp (>90%) | Lost | n/i |
|  | 15_PL2 | Om met | Lost | Overexp (>90%) | Lost | 18.6% |
| **16** | 16_PL2 | Primary Tu | Lost | Overexp (~40%) | Lost | n/i |
|  | 16_PL3 | Liver met | Lost | Overexp (~40%) | Retained | 17.7% |
|  | 16_PL4 | Om met | Lost | Overexp (~30%) | Retained | 15.1% |
| **17** | 17_PL1 | Primary Tu | Lost | Overexp (>75%) | Lost | 32.8% |
|  | 17_PL2 | Liver met | Lost | Overexp (>90%) | Lost | 26.0% |
|  | 17_PL3 | LN met | Lost | Overexp (>90%) | Retained | 35.3% |
|  | 17_PL4 | Om met | Retained | Overexp (~90%) | Lost | 12.1% |
|  | 17_PL5 | AW met | Retained | Overexp (~40%) | Lost | 13.1% |
| **18** | 18_PL2 | Liver met | Retained | Overexp (>75%) | Lost | 31.6% |
| **19** | 19_PL1 | Primary Tu | Retained | Overexp (>90%) | Lost | 11.1% |
|  | 19_PL2 | Primary Tu | Lost | Overexp (~90%) | Lost | 3.5% |
| **20** | 20_PL2 | Primary Tu | Lost | Overexp (~40%) | Lost | 39.4% |
| **21** | 21_PL1 | AW met | Lost | Overexp (>90%) | Retained | 73.3% |
|  | 21_PL2 | Per met | Lost | Overexp (~90%) | Retained | 45.7% |
| **22** | 22_PL2 | Primary Tu | Lost | Retained | Lost | 6.4% |
| **23** | 23_PL1 | Primary Tu | Lost | Overexp (>90%) | Lost | 19.6% |
|  | 23_PL2 | Liver met | Lost | Overexp (>90%) | Lost | 36.2% |
| **24** | 24_PL1 | Om met | Retained | Retained | Retained | 17.7% |
| **25** | 25_PL2 | Primary Tu | Retained | Overexp (~90%) | Lost | 27.7% |
|  | 25_PL3 | Primary Tu | Lost | Overexp (>90%) | Lost | 34.4% |
|  | 25_PL4 | LN met | Lost | Overexp (>90%) | Lost | n/i |
| **26** | 26_PL1 | Primary Tu | Retained | Overexp (~30%) | Retained | 25.4% |
| **27** | 27_PL1 | Primary Tu | Lost | Overexp (~40%) | Lost | 14.8% |
| **28** | 28_PL1 | Primary Tu | Lost | Overexp (>90%) | Lost | 26.9% |
|  | 28_PL2 | Liver met | Lost | Overexp (>90%) | Lost | 48.7% |
| **29** | 29_PL2 | Primary Tu | Lost | Lost | Lost | 18.2% |
| **30** | 30_PL2 | Primary Tu | Retained | Overexp (~70%) | Lost | n/i |
|  | 30_PL3 | LN met | Lost | Overexp (~60%) | Retained | 13.9% |
| **31** | 31_PL1 | Primary Tu | Retained | Overexp (~40%) | Retained | 33.5% |
| **32** | 32_PL1 | Om met | Retained | Lost | Lost | 29.2% |
| **33** | 33_PL2 | Primary Tu | Lost | Overexp (~90%) | Lost | 19.5% |
|  | 33_PL3 | LN met | Retained | Overexp (>90%) | Lost | 18.4% |
| **34** | 34_PL1 | Om met | Lost | Overexp (~90%) | Lost | 26.2% |
| **35** | 35_PL1 | LN met | Lost | Overexp (~80%) | Lost | 39.1% |
| **36** | 36_PL1 | Per met | Lost | Overexp (>90%) | Retained | 24.8% |
| **37** | 37_PL1 | Om met | Lost | Overexp (~90%) | Lost | 35.6% |

AW, abdominal wall; LN, lymph node; met, metastasis; n/i, result not interpretable due to lack of internal control and/or weak staining; Om, omental; Overexp, overexpressed; Per, peritoneum; Tu, tumour; ~, approximately; >, greater than.
