# Supplementary material for: Molecular characteristics of early‐onset pancreatic ductal adenocarcinoma
Source: Mol Oncol. 2024 Jan 3;18(3):677–90. doi: 10.1002/1878-0261.13576 (PMC10920080; doi:10.1002/1878-0261.13576)
Supplement: Supplementary file 7 — Table S6. Reference studies for IHC meta‐synthesis. [file MOL2-18-677-s003.docx]

**Table S6.** Reference studies for IHC meta-synthesis.

| *Age cut-off* | *>50y* | *> 45y* | *≥ 59y* | *>50y* | ***>50y*** |
| --- | --- | --- | --- | --- | --- |
| **IHC in LOPC** | Schlitter et al. 2017 [45] | Hua et al. 2003 [42] | Qian ZR et al. 2018 [46] | Oshima et al 2022 [47] | **Total LOPC** |
| **p16 loss** | 125/172 (72.7%) |  | 240/356 (67.4%) | 61/84 (73%) | 426/612 (69%) |
| **p53 alterations** | 134/172 (78%) |  | 231/356 (65%) | 61/84 (73%) | 426/612 (69%) |
| **SMAD4 loss** | 63/172 (36.7%) | 6/28 (21.4%) | 175/356 (49%) | 38/84 (45%) | 282/640 (44%) |

| *Age cut-off* | *≤40y* | *≤40y* | *≤40y* | *≤45y* | *<50y* | ***<50y*** |
| --- | --- | --- | --- | --- | --- | --- |
| **IHC in EOPC** | Bergmann et al. 2006 [44] | Del Chiaro et al. 2004 [43] | Luttges et al. 2004 [4] | Hua et al. 2003 [42] | Our data | **Total EOPC** |
| **p16 loss** | 7/7 (100%) |  |  |  | 30/35 (86%) | *37/42 (88%)* |
| **p53 alterations** | 5/7 (71%) | 4/6 (66%) | 4/10 (40%) |  | 33/35 (94%) | *46/58 (79%)* |
| **SMAD4 loss** | 6/7 (85.7%) |  |  | 2/6 (33.3%) | 25/35 (71%) | *33/47 (70%)* |

(Reference numbers in brackets refer to the main text list).

**References**

4. Luttges J, Stigge C, Pacena M, Kloppel G. Rare ductal adenocarcinoma of the pancreas in patients younger than age 40 years. *Cancer* 2004;**100**(1):173-82.

42. Hua Z, Zhang YC, Hu XM, Jia ZG. Loss of DPC4 expression and its correlation with clinicopathological parameters in pancreatic carcinoma. *World J Gastroenterol* 2003;**9**(12):2764-7.

43. Del Chiaro M, Menicagli M, Campani D, Funel N, Pollina LE, Decarli N, *et al.* Sporadic pancreatic ductal carcinoma in patient aged less than 40 years. *Pancreas* 2004;**29**(4):336.

44. Bergmann F, Aulmann S, Wente MN, Penzel R, Esposito I, Kleeff J, *et al.* Molecular characterisation of pancreatic ductal adenocarcinoma in patients under 40. *J Clin Pathol* 2006;**59**(6):580-4.

45. Schlitter AM, Jesinghaus M, Jager C, Konukiewitz B, Muckenhuber A, Demir IE, *et al.* pT but not pN stage of the 8th TNM classification significantly improves prognostication in pancreatic ductal adenocarcinoma. *Eur J Cancer* 2017;**84**:121-9.

46. Qian ZR, Rubinson DA, Nowak JA, Morales-Oyarvide V, Dunne RF, Kozak MM, *et al.* Association of Alterations in Main Driver Genes With Outcomes of Patients With Resected Pancreatic Ductal Adenocarcinoma. *JAMA Oncol* 2018;**4**(3):e173420.

47. Oshima M, Okano K, Kamada H, Suto H, Ando Y, Ibuki E, *et al.* P53 immunolabeling in EUS-FNA biopsy can predict low resection rate and early recurrence in resectable or borderline resectable pancreatic cancer treated with neoadjuvant therapy. *J Hepatobiliary Pancreat Sci* 2022;**30**(6):802-14.
